# Testing the Pharmacokinetic Interactions of 24 Colonic Flavonoid Metabolites with Human Serum Albumin and Cytochrome P450 Enzymes

**DOI:** 10.3390/biom10030409

**Published:** 2020-03-06

**Authors:** Violetta Mohos, Eszter Fliszár-Nyúl, Beáta Lemli, Balázs Zoltán Zsidó, Csaba Hetényi, Přemysl Mladěnka, Pavel Horký, Milan Pour, Miklós Poór

**Affiliations:** 1 Department of Pharmacology, Faculty of Pharmacy, University of Pécs, Szigeti út 12, H-7624 Pécs, Hungary; mohos.violetta@gytk.pte.hu (V.M.); eszter.nyul@aok.pte.hu (E.F.-N.); 2 János Szentágothai Research Center, University of Pécs, Ifjúság útja 20, H-7624 Pécs, Hungary; lemli.beata@gytk.pte.hu (B.L.); 3 Institute of Organic and Medicinal Chemistry, Medical School, University of Pécs, Szigeti út 12, H-7624 Pécs, Hungary; 4 Department of Pharmacology and Pharmacotherapy, Medical School, University of Pécs, Szigeti út 12, H-7624 Pécs, Hungary; zsido.balazs@pte.hu (B.Z.Z.); hetenyi.csaba@pte.hu (C.H.); 5 Department of Pharmacology and Toxicology, Faculty of Pharmacy in Hradec Králové, Charles University, Heyrovského 1203, 500 05 Hradec Králové, Czech Republic; mladenkap@faf.cuni.cz (P.M.); 6 Department of Organic and Bioorganic Chemistry, Faculty of Pharmacy in Hradec Králové, Charles University, Akademika Heyrovského 1203, 500 05 Hradec Králové, Czech Republic; horkyp@faf.cuni.cz (P.H.);; 7 Department of Social and Clinical Pharmacy, Faculty of Pharmacy in Hradec Králové, Charles University, Zborovská 2089, 500 05 Hradec Králové, Czech Republic

**Keywords:** polyphenols, colonic flavonoid metabolites, pharmacokinetic interaction, serum albumin, CYP450 enzymes, resorcinol, phloroglucinol, *O*-desmethylangolensin

## Abstract

Flavonoids are abundant polyphenols in nature. They are extensively biotransformed in enterocytes and hepatocytes, where conjugated (methyl, sulfate, and glucuronide) metabolites are formed. However, bacterial microflora in the human intestines also metabolize flavonoids, resulting in the production of smaller phenolic fragments (e.g., hydroxybenzoic, hydroxyacetic and hydroxycinnamic acids, and hydroxybenzenes). Despite the fact that several colonic metabolites appear in the circulation at high concentrations, we have only limited information regarding their pharmacodynamic effects and pharmacokinetic interactions. Therefore, in this *in vitro* study, we investigated the interactions of 24 microbial flavonoid metabolites with human serum albumin and cytochrome P450 (CYP2C9, 2C19, and 3A4) enzymes. Our results demonstrated that some metabolites (e.g., 2,4-dihydroxyacetophenone, pyrogallol, *O*-desmethylangolensin, and 2-hydroxy-4-methoxybenzoic acid) form stable complexes with albumin. However, the compounds tested did not considerably displace Site I and II marker drugs from albumin. All CYP isoforms examined were significantly inhibited by *O*-desmethylangolensin; nevertheless, only its effect on CYP2C9 seems to be relevant. Furthermore, resorcinol and phloroglucinol showed strong inhibitory effects on CYP3A4. Our results demonstrate that, besides flavonoid aglycones and their conjugated derivatives, some colonic metabolites are also able to interact with proteins involved in the pharmacokinetics of drugs.

## 1. Introduction

**Figure 1:**
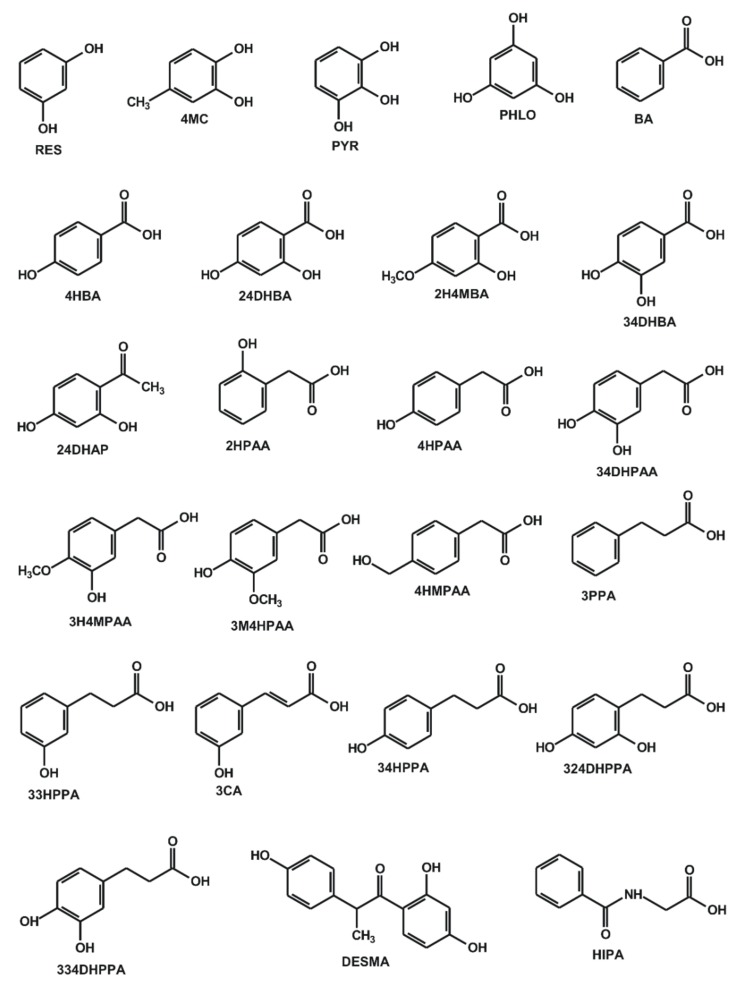
Chemical structures of resorcinol (RES), 4-methylcatechol (4MC), pyrogallol (PYR), phloroglucinol (PHLO), benzoic acid (BA), 4-hydroxybenzoic acid (4HBA), 2,4-dihydroxybenzoic acid (24DHBA), 2-hydroxy-4-methoxybenzoic acid (2H4MBA), 3,4-dihydroxybenzoic acid (34DHBA), 2,4-dihydroxyacetophenone (24DHAP), 2-hydroxyphenylacetic acid (2HPAA), 4-hydroxyphenylacetic acid (4HPAA), 3,4-dihydroxyphenylacetic acid (34DHPAA), 3-hydroxy-4-methoxyphenylacetic acid (3H4MPAA), 3-methoxy-4-hydroxyphenylacetic acid (3M4HPAA), 4-(hydroxymethyl)phenylacetic acid (4HMPAA), 3-phenylpropionic acid (3PPA), 3-(3-hydroxyphenyl)propionic acid (33HPPA), 3-coumaric acid (3CA), 3-(4-hydroxyphenyl)propionic acid (34HPPA), 3-(2,4-dihydroxyphenyl)propionic acid (324DHPAA), 3-(3,4-dihydroxyphenyl)propionic acid (334DHPPA), *O*-desmethylangolensin (DESMA), and hippuric acid (HIPA).

Flavonoids, phenolic compounds found in numerous plants (including fruits and vegetables) [1], have been demonstrated to have beneficial health effects in several *in vitro* and *in vivo* studies [2,3]. Therefore, flavonoid-containing dietary supplements are widely marketed through the Internet. Some of these dietary supplements contain extremely high doses of flavonoids (ranging from several hundreds to thousands of milligrams) [4,5]. Furthermore, flavonoids can interact with proteins involved in drug pharmacokinetics, such as serum albumin, biotransformation enzymes, and drug transporters [6,7,8]. Therefore, the high intake of flavonoids may cause pharmacokinetic interactions with clinically used drugs, as has been reviewed in several papers [9,10,11]. The oral bioavailability of parent flavonoids is low due to their physicochemical properties and high presystemic elimination [12]. In general, flavonoid aglycones are extensively conjugated even in enterocytes and later in hepatocytes, resulting in the production of methyl, sulfate, and glucuronide metabolites [12,13]. A large fraction of flavonoids, not absorbed from the small intestines, can be biotransformed by the colon microbiota, leading to the degradation of flavonoid ring(s) to smaller phenolic compounds. The colonic metabolites can be classified as hydroxybenzoic, hydroxyacetic and hydroxycinnamic acids, and hydroxybenzenes (Figure 1) [14,15,16,17]. Typically, the microbial metabolites of flavonols are phenylacetic and phenylpropionic acids, while flavones and flavanones are biotransformed into phenylpropionic acids (then to benzoic acid) [13]. For example, 3-hydroxyphenylacetic, 3-methoxy-4-hydroxyphenylacetic and 3,4-dihydroxyphenylacetic acids were identified as the major colonic metabolites of quercetin, after the oral administration of quercetin-3-rutinoside to healthy human subjects [13]. As to pharmacokinetic issues, some colonic metabolites were previously shown to interact with serum albumin or biotransformation enzymes, such as pyrogallol (PYR) which form a stable complex with albumin [18], and it is a potent inhibitor of xanthine oxidase enzyme [19,20].

Human serum albumin (HSA) has a major role in the transport of several drugs and xenobiotics in the human circulation [21,22]. There are two major drug binding sites of HSA: the Sudlow’s Site I (subdomain IIA) and Site II (subdomain IIIA) [21]. Displacement of drugs from HSA leads to their elevated free plasma concentrations, which can affect the tissue uptake and/or the speed of elimination of the displaced compound [22]. Cytochrome P450 (CYP) is a superfamily of heme-containing microsomal enzymes [23]. CYP enzymes are crucial in the biotransformation of a wide range of xenobiotics, including drugs and environmental toxins [23,24]. CYP3A4 is the main isoenzyme expressed in the liver and intestines. More than 50% of the orally administered drugs are metabolized by CYP3A4; however, CYP2C9, CYP2C19, CYP2D6, and CYP1A2 are also commonly involved in drug metabolism [25].

Only limited information is available regarding the plasma concentrations of colonic metabolites; however, most of them can be absorbed from the colon. After the high consumption of some fruits (e.g., cranberry), tea, and/or products produced from them, the plasma concentrations of certain microbial flavonoid metabolites can exceed 10 µM [26,27]. These observations suggest that some metabolites may also achieve relevant concentrations in tissues, which is likely enhanced by the extremely high intake of flavonoids through dietary supplements [4].

In this study, we aimed to investigate the interaction of 24 colonic flavonoid metabolites with HSA and CYP (2C9, 2C19, and 3A4) enzymes. HSA-ligand interactions were examined employing fluorescence spectroscopy. Displacement of Site I (warfarin) and Site II (naproxen) markers from HSA by test compounds was evaluated based on ultrafiltration. The inhibitory effects of the metabolites on CYP enzymes were tested *in vitro*, the substrates and the formed metabolites were quantified by high-performance liquid chromatography (HPLC).

## 2. Materials and Methods

### 2.1. Reagents

Human serum albumin (HSA), warfarin, naproxen, testosterone, 6β-hydroxytestosterone, ketoconazole, ticlopidine hydrochloride, CypExpress^TM^ 2C9 kit, CypExpress^TM^ 2C19 kit, CypExpress^TM^ 3A4 kit, resorcinol (RES; benzene-1,3-diol), 4-methylcatechol (4MC; 4-methylbenzene-1,2-diol), pyrogallol (PYR; benzene-1,2,3-triol), phloroglucinol (PHLO; 1,3,5-trihydroxybenzene), benzoic acid (BA), 4-hydroxybenzoic acid (4HBA), 2,4-dihydroxybenzoic acid (24DHBA; 4-hydroxysalicylic acid), 2-hydroxy-4-methoxybenzoic acid (2H4MBA; 4-methoxysalicylic acid), 3,4-dihydroxybenzoic acid (34DHBA; protocatechuic acid), 2,4-dihydroxyacetophenone (24DHAP), 2-hydroxyphenylacetic acid (2HPAA), 4-hydroxyphenylacetic acid (4HPAA), 3,4-dihydroxyphenylacetic acid (34DHPAA), 3-hydroxy-4-methoxyphenylacetic acid (3H4MPAA), 3-methoxy-4-hydroxyphenylacetic acid (3M4HPAA; homovanillic acid), 4-(hydroxymethyl)phenylacetic acid (4HMPAA), 3-phenylpropionic acid (3PPA; hydrocinnamic acid), 3-coumaric acid (3CA; 3-hydroxycinnamic acid), 3-(4-hydroxyphenyl)propionic acid (34HPPA; phloretic acid), 3-(2,4-dihydroxyphenyl)propionic acid (324DHPAA; 3,4-dihydroxy-hydrocinnamic acid), hippuric acid (HIPA; benzoylaminoacetic acid) were obtained from Sigma-Aldrich (St. Louis, MO, US). 3-(3-hydroxyphenyl)propionic acid (33HPPA) and 3-(3,4-dihydroxyphenyl)propionic acid (334DHPPA; 3,4-dihydroxyphenylcinnamic acid) were purchased from Toronto Research Chemicals (North York, Ontario, Canada). Racemic *O*-desmethylangolensin (DESMA; 1-(2,4-dihydroxyphenyl)-2-(4-hydroxyphenyl)-1-propanone) was synthetized as described previously [28]. S-mephenytoin, 4-hydroxymephenytoin, diclofenac, 4′-hydroxydiclofenac, and sulfaphenazole were obtained from Carbosynth (Berkshire, UK). Flavonoid metabolites were dissolved in dimethyl sulfoxide (DMSO; Fluka, Bucharest, Romania), and stock solutions (10 mM) were stored at −20 °C.

### 2.2. Spectroscopic Measurements

Albumin-ligand interactions were investigated employing a Hitachi F-4500 fluorimeter (Tokyo, Japan), measurements were performed in phosphate-buffered saline (PBS, pH 7.4), in the presence of air at room temperature. Absorption spectra of the flavonoid metabolites were also recorded in PBS, applying a HALO DB-20 UV-Vis spectrophotometer (Dynamica, London, UK). Because the inner filter effect can decrease the fluorescence emission signal of albumin, fluorescence spectra were corrected using the following equation [29,30]:

I_cor_ = I_obs_ × e^(A^_ex_^+ A^_em_^)/2^ (1)

where *I_cor_* means the corrected and *I_obs_* denotes the observed emission intensities at the wavelengths used, while *A_ex_* and *A_em_* are the absorbance values of flavonoid metabolites at the excitation and emission wavelengths applied, respectively.

HSA-ligand interactions were evaluated using fluorescence quenching studies or the intrinsic fluoresce of the metabolite (if it strongly interfered with the emission signal of albumin). In quenching studies, the emission spectrum of HSA (2 μM) was recorded in the presence of increasing concentrations of microbial metabolites (0, 2, 3, 4, 5, 6, and 8 μM), using a 295 nm excitation wavelength. Data were evaluated based on linear and non-linear fitting, employing the Stern-Volmer equation (Equation (2)) and the Hyperquad2006 program package (Protonic Software; Leeds, UK) [30,31], respectively. The Stern-Volmer equation was described as

I_0_/I = 1 + K_SV_ × [Q] (2)

where *I_0_* and *I* denote the fluorescence emission intensities (λ_ex_ = 295 nm, λ_em_ = 340 nm) of HSA in the absence and presence of colonic metabolites, respectively. Furthermore, *K_SV_* (unit: L/mol) and *[Q]* (unit: mol/L) are the Stern-Volmer quenching constant and the concentration of the quencher, respectively.

Since 2H4MBA showed strong fluorescence at the emission maximum of HSA (340 nm), the interaction of 2H4MBA with albumin was investigated based on the increase in its emission signal in the presence of HSA at 395 nm. The fluorescence emission spectrum of 2H4MBA (2 μM) was recorded with HSA (0, 0.5, 1, 2, 3, 4 and 5 μM), using 295 nm excitation wavelength (the excitation maximum of 2H4MBA). The binding constants (*K*; unit: L/mol) of albumin-ligand complexes were determined by non-linear fitting using the Hyperquad2006 program, as has been reported [30,31]. During this evaluation, absorbance values and fluorescence signals of both HSA and 2H4MBA were taken into account.

### 2.3. Ultrafiltration Experiments

The displacing effects of colonic flavonoid metabolites vs. Site I marker warfarin and Site II marker naproxen were examined based on ultrafiltration, employing the previously described methods [32,33]. Briefly, samples containing warfarin with HSA (1 and 5 μM, respectively) or naproxen with HSA (1 and 1.5 μM, respectively) both without and with flavonoid metabolites (20 μM) were filtered, employing Pall Microsep Advance centrifugal devices (30 kDa molecular weight cut-off value; VWR, Budapest, Hungary). Then the concentrations of site markers in the filtrates were quantified by HPLC (see in 2.5).

### 2.4. CYP Assays

The inhibitory effects of colonic flavonoid metabolites were examined *in vitro*, applying CypExpress^TM^ Cytochrome P450 human kits. In each assay, FDA-recommended substrates (CYP2C9: diclofenac; CYP2C19: S-mephenytoin; CYP3A4: testosterone) and positive controls (CYP2C9: sulfaphenazole; CYP2C19: ticlopidine; CYP3A4: ketoconazole) were used as well as solvent controls.

Inhibition of CYP2C9 enzyme was examined based on CYP2C9-catalyzed 4′-hydroxydiclofenac formation in the presence of microbial metabolites, using the previously reported method with some modifications [32,33]. Briefly, each incubate (with a 200-μL final volume) contained diclofenac (5 μM; substrate) and CypExpress^TM^ 2C9 kit (6 mg/mL; including the NADPH generating system), in the presence of test compounds (0–30 μM) or the positive control (sulfaphenazole), in 0.05 M potassium phosphate buffer (pH 7.5). The incubations were started with the addition of the enzyme. After 120 min incubation at 700 rpm and 30 °C in a thermomixer (Eppendorf, Hamburg, Germany), the reaction was stopped with the addition of 100 μL of ice-cold methanol. After centrifugation (10 min, 14,000 g, room temperature), the concentrations of diclofenac and 4′-hydroxydiclofenac were quantified with HPLC (see in 2.5).

Effects of microbial metabolites on CYP2C19 enzyme were examined based on their impact on CYP2C19-catalyzed 4-hydroxymephenytoin formation, employing the previously reported method without modifications [33].

Inhibitory action of flavonoid metabolites on CYP3A4 enzyme was tested based on their effects on CYP3A4-catalyzed testosterone hydroxylation, using the previously described method without modifications [32,33].

The metabolite formation (% of control) was depicted vs. the concentrations of inhibitors in a decimal logarithmic scale, then IC_50_ values were determined employing the GraphPad Prism 8 software (San Diego, CA, USA).

### 2.5. HPLC Analyses

The following HPLC system was used to quantify site markers as well as substrates and products of CYP enzymes: Waters 510 pump (Milford, MA, USA), Rheodyne 7125 injector (Berkeley, CA, USA) with a 20-μL sample loop, Waters 486 UV detector (Milford, MA, USA), and Jasco FP-920 fluorescence detector (Tokyo, Japan). The data were evaluated using Waters Millennium Chromatography Manager (Milford, MA, USA).

After ultrafiltration, warfarin and naproxen (Site I and II markers of HSA, respectively) concentrations in the filtrates were quantified directly, using the previously described methods without modifications [32,33], by fluorescence and UV detectors, respectively.

Diclofenac and 4′-hydroxydiclofenac (CYP2C9 assay) were quantified by HPLC-UV as it was reported previously [32,33]. However, DESMA was co-eluted with the substrate; therefore, the following HPLC method was used to determine its inhibitory effect on CYP2C9 enzyme. The separation was carried out using a guard column (Phenomenex Security Guard C8, 4.0 × 3.0 mm) linked to an analytical column (Agilent Eclipse C8, 150 × 4.6 mm, 5 μm) at room temperature. The mobile phase contained sodium phosphate buffer (10 mM, pH 4.55) and acetonitrile (60:40 v/v%), and was driven through the column with 1.0 mL/min (0–7.5 min) then with 1.2 mL/min (7.5–13 min) flow rate. Diclofenac and 4′-hydroxydiclofenac were detected at 275 nm.

S-mephenytoin and 4-hydroxymephenytoin (CYP2C19 assay) as well as testosterone and 6β-hydroxytestosterone (CYP3A4 assay) were quantified by HPLC-UV employing the previously described methods without modifications [32,33].

### 2.6. Modeling Studies

Ligand structures of DESMA, RES, and PHLO were built in Maestro [34] and energy-minimized using quantum chemistry program package, MOPAC [35] with a PM7 parametrization [36] and a gradient norm set to 0.001. Force calculations were also performed using MOPAC, the force constant matrices were positive definite. Gasteiger-Marsilli partial charges were assigned in AutoDock Tools [37]. Flexibility was allowed on the ligand at all active torsions. These prepared structures were used for docking. In the IUPAC name of *O*-desmethylangolensin (1-(2,4-dihydroxyphenyl)-2-4-hydroxyphenyl)propan-1-one), there is no information about chirality. Based on previous studies, dominantly *R*(-)-DESMA is produced (approximately 90%) in the human body [38,39,40]. In the present study, we docked both the *R*- and *S*- configurations of this ligand.

The apo structures of CYP3A4 were thoroughly investigated to select the target. For CYP2C9 only one apo structure was available. Atomic coordinates of the targets were obtained from the Protein Data Bank (PDB). Apo structures of CYP3A4 (PDB code 5vcc) and CYP2C9 (PDB code 1og2) were used for docking of ligands DESMA, RES, and PHLO. The holo structure of CYP2C9 (PDB code 1og5) used to test the applicability of the method, the original crystallographic ligand *S*-warfarin was redocked onto the target.

Atomic partial charges of heme were adopted as the ferric penta coordinate high spin charge model from reference [41]. The rest of the target molecule was equipped with polar hydrogen atoms and Gasteiger-Marsilli partial charges in AutoDock Tools as in our previous study [42].

All ligand structures were docked to the active site of the enzymes located above the heme ring using AutoDock 4.2.6 [37]. The number of grid points was set to 90×90×90 at a 0.375 Å grid spacing. Lamarckian genetic algorithm was used for global search, with the flexibility of all active torsions allowed on the ligand. Ten docking runs were performed, and the resultant ligand conformations were ranked by their free binding energy values. Representative docked ligand conformations were used to collect interacting target amino acid residues with a 3.5 Å cut-off distance calculated for heavy atoms. Root mean squared deviation (RMSD) values were calculated between the heavy atoms of the crystallographic and representative ligand conformations.

### 2.7. Statistics

Data demonstrate mean and standard error of the mean (±SEM) values, derived at least from three independent experiments. Statistical significance (p < 0.05 and p < 0.01) was evaluated based on one-way ANOVA test followed by Tukey’s post-hoc test (IBM SPSS Statistics, Version 21; Armonk, NY, USA).

## 3. Results

### 3.1. Interaction of Colonic Flavonoid Metabolites with Human Serum Albumin Determined by Fluorescence Spectroscopy

**Figure 2:**
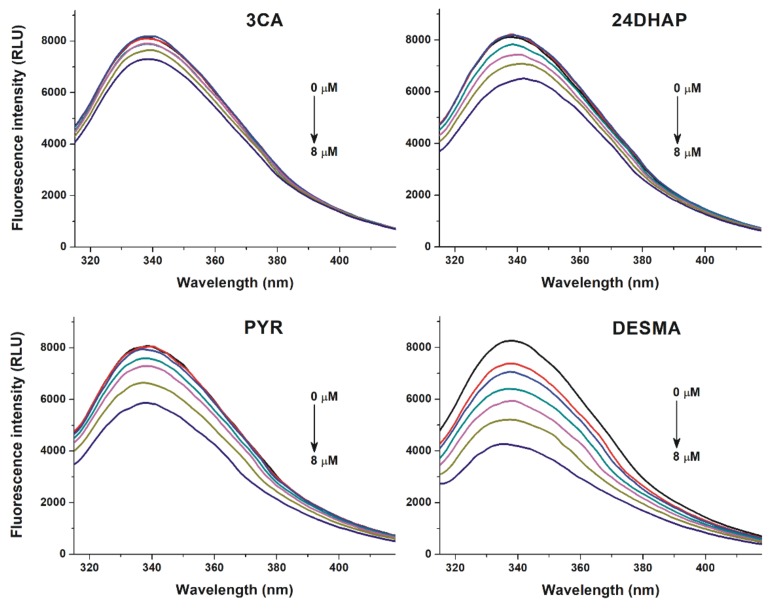
Fluorescence quenching effects of colonic flavonoid metabolites. Representative emission spectra of HSA (2 μM) in the presence of increasing concentrations (0, 2, 3, 4, 5, 6, and 8 µM) of 3-coumaric acid (3CA), 2,4-dihydroxyacetophenon (24DHAP), pyrogallol (PYR), and *O*-desmethylangolensin (DESMA) in PBS (pH 7.4; λ_ex_ = 295 nm).

**Figure 3:**
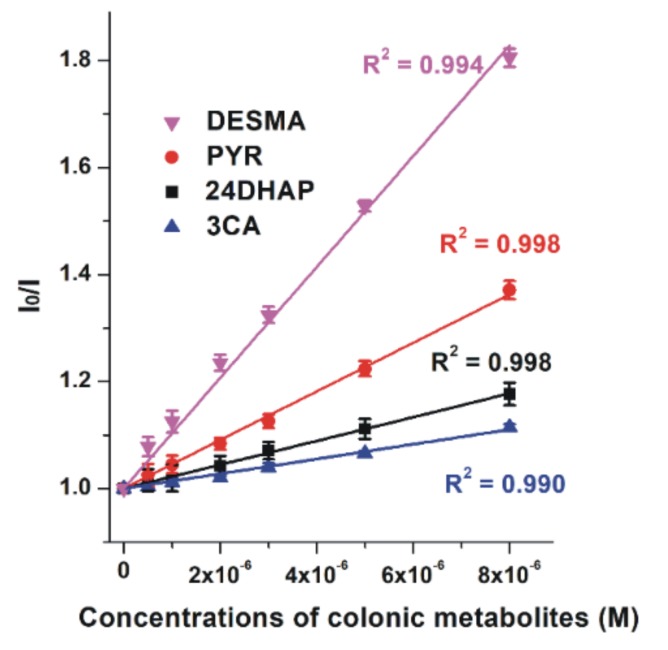
Stern-Volmer plots of metabolite-HSA complexes (λ_ex_ = 295 nm, λ_em_ = 340 nm). Means and SEM values are derived from three independent experiments (DESMA, *O*-desmethylangolensin; PYR, pyrogallol; 24DHAP, 2,4-dihydroxyacetophenon; 3CA, 3-coumaric acid).

To investigate the potential interactions of colonic metabolites with HSA, fluorescence quenching experiments were performed. Among the 24 metabolites tested, 3CA, 24DHAP, PYR, and DESMA caused significant decrease in HSA fluorescence signal at 340 nm (λ_ex_ = 295 nm; Figure 2). DESMA induced the strongest decrease in the emission of HSA, followed by PYR, 24DHAP, and 3CA. The quenching effects of colonic metabolites on HSA was evaluated employing the Stern-Volmer equation (Equation (2)). Stern-Volmer plots and decimal logarithmic *K_SV_* values are demonstrated in Figure 3 and Table 1, respectively. After the elimination of inner-filter effects of compounds tested, Stern-Volmer plots showed excellent linearity (R^2^ = 0.990–0.998), suggesting the static quenching effects of 3CA, 24DHAP, PYR, and DESMA on the fluorescence signal of HSA.

**Figure 4:**
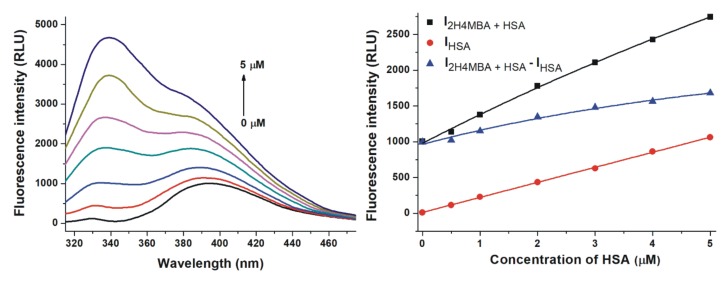
Effect of human serum albumin (HSA) on the fluorescence emission signal of 2-hydroxy-4-methoxybenzoic acid (2H4MBA). Left: Representative emission spectra of 2H4MBA (2 μM) in the presence of increasing concentrations of HSA (0, 0.5, 1, 2, 3, 4 and 5 μM) in PBS (pH 7.4). Right: Fluorescence emission intensity of 2H4MBA in the presence of increasing concentrations of HSA (I_2H4MBA+HSA_), HSA alone (I_HSA_), and I_2H4MBA+HSA_ – I_HSA_ in PBS (pH 7.4; λ_ex_ = 295 nm, λ_em_ = 395 nm).

2H4MBA exerted significant fluorescence at 340 nm which interfered with the emission signal of HSA (Figure 4). Therefore, emission spectra of 2H4MBA (2 μM) in the presence of increasing HSA concentrations (0-5 μM; Figure 4, left) and the same concentrations of HSA alone (without 2H4MBA) were also recorded (λ_ex_ = 295 nm). Data were evaluated at the emission wavelength maximum of 2H4MBA (395 nm), where the observed fluorescence signal highly exceeded the sum of the emission signals of 2H4MBA and HSA (Figure 4, right). This suggests the formation of 2H4MBA-HSA complexes.

Based on quenching studies (Figure 2) and the HSA-induced increase in the fluorescence of 2H4MBA (Figure 4), the binding constants (*K*) of albumin-ligand complexes were determined by non-linear fitting, employing the Hyperquad2006 software [30,31]. Table 1 demonstrates the decimal logarithmic *K* values, suggesting that 2H4MBA and DESMA binds to albumin with the highest affinity among the compounds tested. Log*K* and log*K_SV_* values showed excellent correlation.

### 3.2. Displacement of Site I and II Markers from Human Serum Albumin Determined by Ultrafiltration

**Figure 5:**
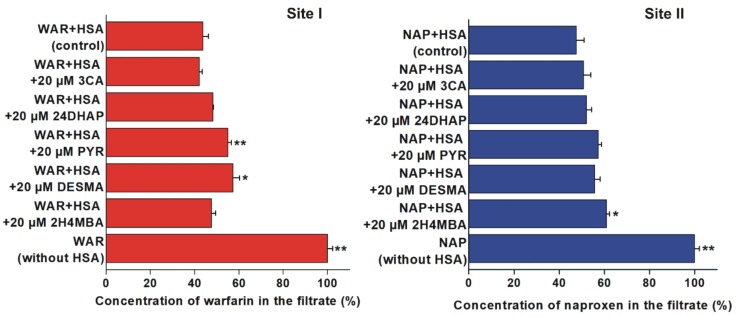
Displacement of warfarin (WAR; left) and naproxen (NAP; right) from HSA based on ultrafiltration (*p < 0.05, **p < 0.01). Left: Concentration of WAR in the filtrate after ultrafiltration in the absence and presence of 3-coumaric acid (3CA), 2,4-dihydroxyacetophenon (24DHAP), pyrogallol (PYR), *O*-desmethylangolensin (DESMA), and 2-hydroxy-4-methoxybenzoic acid (2H4MBA) (WAR: 1 µM; HSA: 5 µM). Right: Concentration of NAP in the filtrate after ultrafiltration in the absence and presence of 3CA, 24DHAP, PYR, DESMA, and 2H4MBA (NAP: 1 µM; HSA: 1.5 µM). Means and SEM values presented are derived from three independent experiments.

To test the displacing ability of 3CA, 24DHAP, PYR, DESMA, and 2H4MBA vs. the Site I marker warfarin and the Site II marker naproxen, ultrafiltration experiments were performed. PYR (p < 0.01) and DESMA (p < 0.05) significantly increased the concentration of warfarin in the filtrate (Figure 5, left). Furthermore, 2H4MBA was the sole compound which induced statistically significant (p < 0.05) but weak elevation of filtered naproxen concentration (Figure 5, right).

### 3.3. Inhibition of CYP2C9 Enzyme by Colonic Flavonoid Metabolites

**Figure 6:**
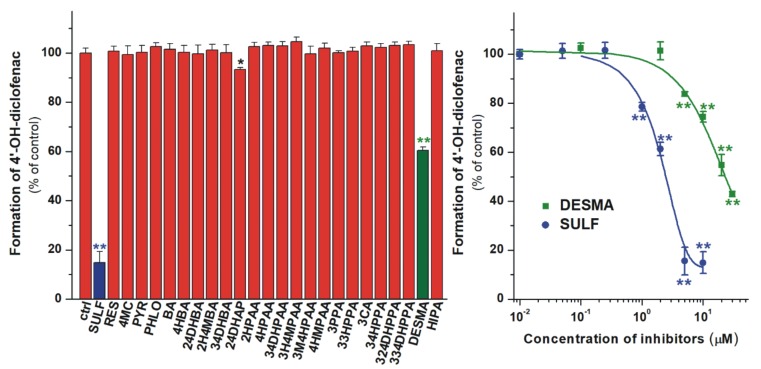
Left: Inhibitory effects of colonic flavonoid metabolites and sulfaphenazole (SULF, positive control; 20 μM each) on the CYP2C9-catalyzed diclofenac (5 μM) hydroxylation. *O*-desmethylangolensin (DESMA) was the sole compound which considerably inhibited the enzyme. Right: Concentration-dependent inhibition of CYP2C9 by DESMA and SULF (* p < 0.05; ** p < 0.01). Means and SEM values demonstrated are derived from three independent experiments.

In the following experiments, the inhibitory effects of the microbial flavonoid metabolites on the CYP2C9-catalyzed diclofenac hydroxylation were examined. Because of the high number of test compounds, in the first experiments, their four-fold concentration (20 μM) vs. the substrate was investigated. Among the 24 substances tested, only 24DHAP and DESMA significantly inhibited the enzyme. 24DHAP caused only a slight inhibition, while DESMA decreased the metabolite formation even at low micromolar concentrations (Figure 6). Despite the strong inhibitory effect of DESMA, it showed considerably (five-fold) weaker effect than the positive control sulfaphenazole (Figure 6, right; Table 2).

### 3.4. Inhibition of CYP2C19 Enzyme by Colonic Flavonoid Metabolites

**Figure 7:**
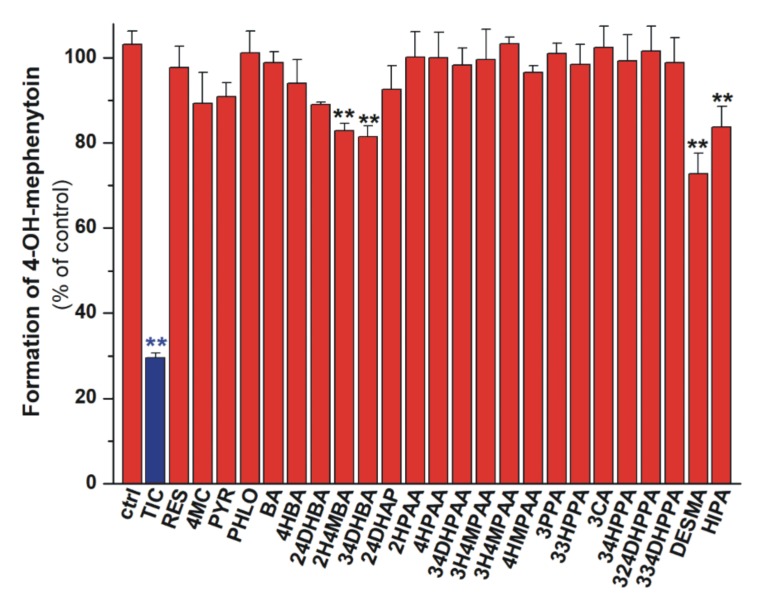
Inhibitory effects of colonic flavonoid metabolites and ticlopidine (TIC, positive control; 20 μM each) on the CYP2C19-catalyzed S-mephenytoin (5 μM) hydroxylation. 2-hydroxy-4-methoxybenzoic acid (2H4MBA), 3,4-dihydroxybenzoic acid (34DHBA), hippuric acid (HIPA), and *O*-desmethylangolensin (DESMA) caused statistically significant (** p < 0.01) but only weak inhibition of the enzyme. Means and SEM values presented are derived from three independent experiments.

We also tested the effects of microbial metabolites on CYP2C19-catalyzed S-mephenytoin hydroxylation. Our results demonstrated that 2H4MBA, 34DHBA, HIPA, and DESMA induced statistically significant decrease in the metabolite formation at four-fold concentrations (20 μM) vs. the substrate (Figure 7). However, even these compounds induced less than 30% inhibition, and proved to be considerably weaker inhibitors than the positive control ticlopidine.

### 3.5. Inhibition of CYP3A4 Enzyme by Colonic Flavonoid Metabolites

**Figure 8:**
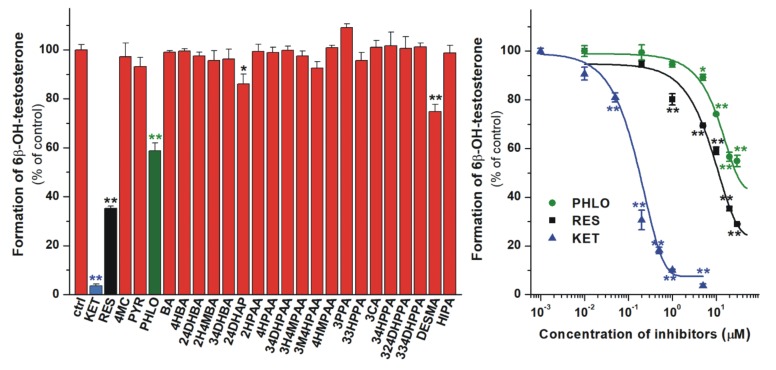
Left: Inhibitory effects of colonic flavonoid metabolites and ketoconazole (KET, positive control; each 20 μM) on the CYP3A4-catalyzed testosterone (5 μM) hydroxylation. 2,4-dihydroxyacetophenone (24DHAP), *O*-desmethylangolensin (DESMA), phloroglucinol (PHLO), and resorcinol (RES) caused statistically significant decrease in metabolite formation. Right: Concentration-dependent inhibition of CYP3A4 by PHLO, RES, and KET (* p < 0.05; ** p < 0.01). Means and SEM values demonstrated are derived from three independent experiments.

The inhibitory effects of microbial metabolites on CYP3A4-catalyzed testosterone hydroxylation were also examined. At four-fold concentrations (20 μM) vs. the substrate, 24DHAP and DESMA showed less than 30% inhibition of CYP3A4 (Figure 8, left). However, PHLO and RES exerted stronger inhibitory effect on the enzyme, causing approximately 40% and 65% decrease in metabolite formation under the same circumstances. The concentration-dependent inhibitory actions of PHLO, RES, and the positive control ketoconazole are demonstrated in Figure 8 (right), showing that PHLO and RES are approximately 20- and 30-fold weaker inhibitors of CYP3A4 than ketoconazole, respectively (Table 2).

### 3.6. Modeling Studies

Docking of *S*-warfarin served as a test of applicability of the methodology for producing close-to crystallographic bound ligand conformation. The known heme-bound crystallographic ligand conformation of *S*-warfarin was used as a reference for checking the applicability of our computational docking protocol for atomic resolution calculation of the ligand binding mode. Re-docking of *S*-warfarin into the holo CYP2C9 structure (PDB code 1og5) was successful, and the crystallographic ligand binding mode was reproduced at an RMSD value of 1.24 Å in the top 1^st^ rank. Docking of *S*-warfarin was also performed for the apo CYP2C9 (PDB code 1og2) structure at an RMSD value of 4.08 Å (top 3^rd^ rank).

Ligands DESMA, PHLO, and RES were docked to the active sites of both apo enzyme structures (PDB codes 5vcc and 1og2 for CYP3A4 and CYP2C9, respectively). In the cases of the query ligands, the binding modes are de novo described in the present study.

**Figure 9:**
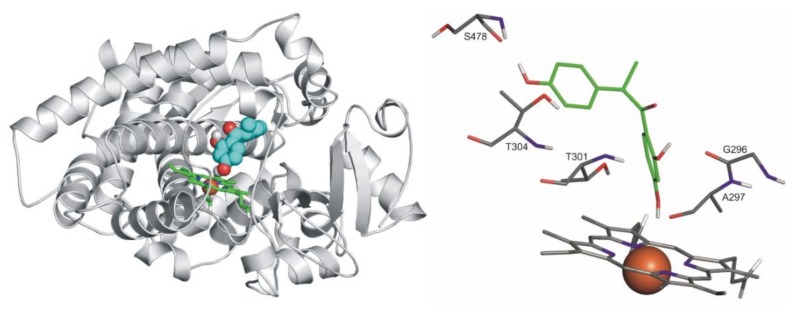
Left: The binding mode of *R*-DESMA (space-filling) as docked to the binding pocket of CYP2C9 enzyme (teal cartoon) above the heme ring (green sticks). Right: The close-up of binding mode of *R*-DESMA (green sticks) as docked to the binding pocket of CYP2C9 enzyme above the heme ring (Fe^3+^ ion in orange). Interacting enzyme residues are labelled and shown as thin sticks. Interacting amino acids: G296, A297, E300, T301, T304, and L361; binding energy: −5.93 kcal/mol.

Both *R*- and *S*-enantiomers of DESMA were docked. Notably, the *R*-enantiomer is the dominant metabolite of daidzein (approximately 90% *R*- vs. 10% *S*-enantiomer is formed) [38,39,40]. Interestingly, the *S*-enantiomer only found binding positions that appeared to be prerequisite modes if compared to the *R*-enantiomer. The latter found a position appearing to be a final binding mode, due to its unambiguous coordination to the heme iron (Figure 9). Likewise ketoconazole (a well-known inhibitor of CYP3A4), where the imidazole N atom of ketoconazole coordinates to the heme iron with a distance of 2.70 Å [42], this binding mode of the *R*-DESMA was reached in the top 3^rd^ rank with the O atom of its hydroxyl group being of 3.60 Å distance from the heme iron (regarding CYP2C9). G296, T301, T304 and S478 amino acids of the enzyme form H-bonds with the ligand.

**Figure 10:**
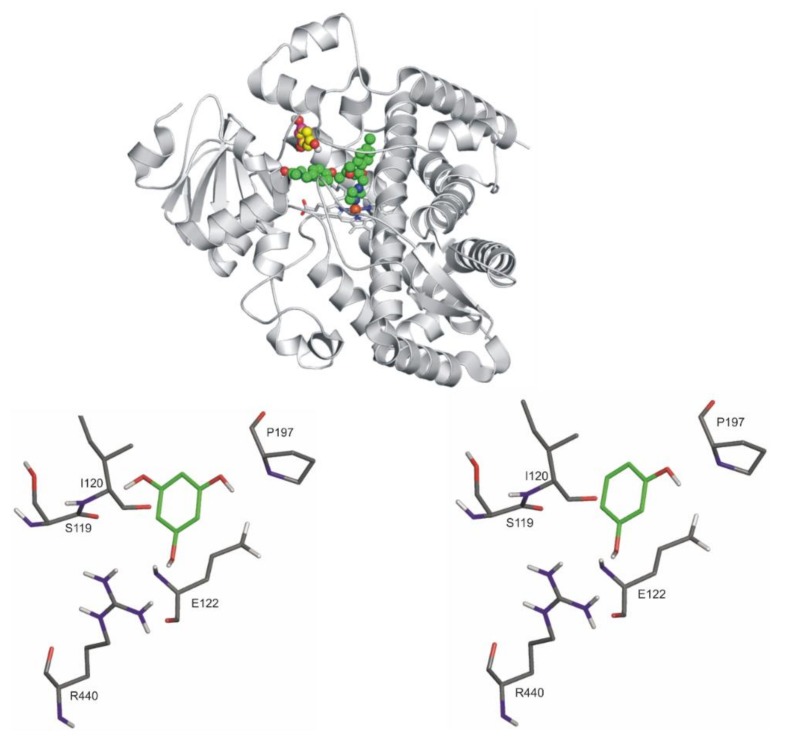
Top: The binding mode of ketoconazole (space-filling, green) superimposed from the crystallographic image (PDB code 2v0m) and the ligands, PHLO and RES (space filling, yellow, and pink, respectively) as docked to the binding pocket of CYP3A4 enzyme (teal cartoon) above the heme ring (teal sticks, iron with orange sphere). Notably, the binding positions of PHLO and RES overlap significantly; therefore, they cannot be demonstrated separately. Bottom, left: The close-up of binding mode of PHLO (green sticks) as docked to the binding pocket of CYP3A4 enzyme. Interacting enzyme residues are labelled and shown as thin sticks. Bottom, right: The close-up of binding mode of RES (green sticks) as docked to the binding pocket of CYP3A4 enzyme. Interacting enzyme residues are labeled and shown as thin sticks. Interacting amino acids (both RES and PHLO): N104, R105, R106, P107, F108, S119, I120, A121, E122, and R440; binding energies: −6.26 kcal/mol (RES) and −6.46 kcal/mol (PHLO).

The docking of both PHLO and RES resulted in one binding position for each ligand (Figure 10, top). The binding positions of both compounds are shown separately compared to ketoconazole (a well-known inhibitor of CYP3A4) (Figure 10, PHLO: bottom left, RES: bottom right). These positions are more than 10 Å distance from the heme iron, overlapping significantly. S119, E122, P197, and R440 amino acid residues of the enzyme stabilize the binding of both ligands through H bonds.

## 4. Discussion

As it has been reported, flavonoids and their conjugated metabolites interact with serum albumin [5,43,44] and with several biotransformation enzymes [45,46,47,48]. Flavonoids and their conjugated metabolites reach only nanomolar or low micromolar systemic plasma concentrations [49], in contrast to some of the colonic flavonoid metabolites [27]. However, there are only limited data regarding the effects of colonic flavonoid metabolites on proteins involved in pharmacokinetics of drugs. Therefore, in this study, we investigated the interactions of 24 colonic flavonoid metabolites with HSA and CYP (2C9, 2C19, and 3A4) enzymes.

3CA, 24DHAP, PYR, and DESMA decreased the fluorescence emission signal of HSA at 340 nm (Figure 2). Since the inner-filter effects of the metabolites were corrected (Figure S1; Equation (1)), these observations show that 3CA, 24DHAP, PYR, and DESMA can partly quench the intrinsic fluorescence of the Trp-214 residue in HSA, in a concentration-dependent fashion. Therefore, it is reasonable to hypothesize the formation of albumin-ligand complexes [48,50]. Furthermore, HSA strongly increased the fluorescence emission signal of 2H4MBA at 395 nm (Figure 4). Water molecules can partly quench the intrinsic fluorescence of aromatic fluorophores [51]. The interaction of these ligand molecules with albumin results in the partial decomposition of their hydration shell, which consequently leads to the increase in their fluorescence signal. Based on these principles, 2H4MBA also interacts with HSA. The binding constants were calculated employing non-linear fitting with the Hyperquad software (Table 1). Our results suggest that 2H4MBA and DESMA form highly stable complexes with HSA (log*K* ≈ 5.1), that are comparable to the binding constant of warfarin-HSA complex (log*K* = 5.3) [52]. Thus, the albumin-binding may be an important factor in their pharmacokinetics. Lower stability of PYR-HSA and 24DHAP-HSA complexes were determined, while 3CA formed poorly stable complexes with HSA (log*K* = 4.1).

The interactions of some compounds tested have been examined in previous studies [18,53,54,55,56]. The log*K* value of phenylacetic acid-HSA complex is between 3 and 4 [57,58], and 3M4HPAA (its hydroxy-methoxy-substituted derivative) has a similar affinity toward bovine serum albumin (log*K* = 3.8) [53]. HIPA (log*K* = 3.8), 4-HBA (no interaction), 24DHBA (log*K* = 3.2), and 34DHBA (log*K* = 3.1) formed no or poorly stable complexes with HSA [18,54,55,56], which explains why we did not observe their relevant interactions with albumin in quenching studies. Our results show high affinity of 2H4MBA toward HSA (log*K* = 5.1); however, a recent study suggests a considerably lower stability of 2H4MBA-HSA complex (log*K* = 3.2) [55]. It may result from the fluorescence emission spectra of 2H4MBA, overlapping with the emission maximum of HSA (Figure 4), disrupting the precise determination of the binding constant in a quenching study (performed Zhang et al.) [55]. Previously reported log*K* values of 3CA-bovine serum albumin (log*K* = 4.1) [56] and PYR-HSA (log*K* = 4.5) [18] complexes are in a good agreement with our results.

3CA achieves only low nanomolar concentrations in the circulation [27]. We did not find plasma concentration data regarding 24DHAP; however, it appeared in the circulation after the administration of Liu Wei Di Huang Wan (traditional Chinese herbal drug) to rats [59]. After the consumption of mixed berry fruit purée by healthy human volunteers, the concentration of PYR sulfates exceeded 10 μM in plasma samples [60]. Furthermore, after the consumption of black tea extract (2650 mg), approximately 7 μM peak plasma concentration of PYR was observed in healthy human subjects (importantly: plasma samples were not treated with sulfatase) [26]. Plasma concentrations of DESMA, after the dietary intake or high consumption of soy products, show great variability with low nanomolar values to 0.5 μM [38,61]. After the administration of *Decalepis arayalpathra* tuber extract to rats, high concentrations of 2H4MBA (C_max_ = 7.7 μM) were quantified in rat plasma [62]. Furthermore, the orally-administered 4-hydroxy-3-methoxy-benzoic acid (structural isomer of 2H4MBA) was absorbed and produced micromolar concentrations (C_max_ = 5.7 μM) in healthy human volunteers [26]. These data underscore that the albumin-binding of 24DHAP, PYR, DESMA, and 2H4MBA may have biological and/or pharmacological importance.

In ultrafiltration studies, warfarin concentration was increased by PYR and DESMA, while naproxen concentration was elevated by 2H4MBA in the filtrates (Figure 5). Since HSA is a large macromolecule (66.5 kDa), HSA and albumin-bound molecules cannot pass through a filter with 30 kDa molecular weight cut-off value. Therefore, the increase in the concentration of site markers in the filtrate indicates their displacement from HSA [33]. Considering these principles and our observations, it is reasonable to hypothesize that PYR and DESMA displaced warfarin from Site I, and 2H4MBA displaced naproxen from Site II. Nevertheless, the fact that 20 μM concentrations of flavonoid metabolites (vs. 1 μM site marker concentrations) induced no or only slight displacement of warfarin and naproxen suggests their poor displacing ability vs. both sites. In the same experimental models used, several flavonoids (with log*K* values in the range of 5.2-5.7) caused significantly stronger displacement of warfarin (quercetin, quercetin-3′-sulfate, isorhamnetin, tamarixetin, chrysin, chrysin-7-sulfate, and 7,8-dihydroxyflavone) and/or naproxen (chrysin, chrysin-7-sulfate, and 7,8-dihydroxyflavone) [5,33,48] compared to the colonic flavonoid metabolites tested in this study. Therefore, it seems to be unlikely that these microbial metabolites can disrupt the albumin binding of drugs.

In our experiments, DESMA was the sole metabolite tested which strongly inhibited CYP2C9 (Figure 6). In agreement with our observations, 4HBA and 34HPPA did not inhibit [63] while HIPA inhibited only at very high concentrations (e.g., 100 μM) [64] the CYP2C9 enzyme in previous studies. As it has been reported, the soy isoflavone daidzein inhibits the CYP2C9-catalyzed hydroxylation of diclofenac [65]. Furthermore, in the human intestinal tract, a significant amount of daidzein is biotransformed to DESMA [66]. Therefore, it is reasonable to hypothesize that DESMA may further increase the inhibitory effect of daidzein on CYP2C9 *in vivo*. Based on previously reported data, the peak plasma concentration of DESMA (after the ingestion of 60 g baked soybean powder) can reach 300−500 nM [61].

Under the applied conditions, the statistically significant but weak inhibition of CYP2C19 by 2H4MBA, 34DHBA, HIPA, and DESMA was observed (Figure 7). We did not find any data in the scientific literature regarding the effects of these compounds on CYP2C19. Qiao et al. reported that 4HBA and 34HPPA did not inhibit the CYP2C19 enzyme [63], in accordance with our study. Based on these results, it seems to be unlikely that 2H4MBA, 34DHBA, HIPA, and DESMA can cause relevant interaction with drugs biotransformed by CYP2C19.

Among the 24 metabolites tested, 24DHAP, DESMA, PHLO, and RES significantly inhibited the CYP3A4 enzyme (Figure 8). Based on our current knowledge, the inhibitory effects of these compounds on CYP3A4 have not been reported. In previous studies, HIPA did not show relevant inhibition at 1-100 μM range [64] or exerted considerable inhibition only above 50 μM concentrations [67] on the CYP3A4-catalyzed testosterone hydroxylation. In another studies, 4HBA and 34HPPA [63,68,69] as well as 4-coumaric acid (the structural isomer of 3CA) [70] did not influence CYP3A4 enzyme. Thus, our results are consistent with the previously reported data. Despite the fact that RES and PHLO are much weaker inhibitors of CYP3A4 than ketoconazole, they caused similarly strong inhibition of the enzyme in the same experimental model as bergamottin did [42] (which shows clinically relevant interaction with CYP3A4). Therefore, the effects of RES and PHLO on CYP3A4 may cause relevant pharmacokinetic interactions, while the inhibitory effect of 24DHAP and DESMA does not seem considerable. Based on previous investigations, PHLO can be absorbed and reach the systemic circulation: after the oral administration of 160 mg PHLO, approximately 5 μM peak plasma concentrations were determined in healthy human volunteers [71,72]. However, we did not find any data regarding the plasma concentrations of RES.

Some colonic flavonoid metabolites (e.g., 33HPPA, 34DHPAA, 4-MC, and DESMA) may contribute to the positive cardiovascular effects of flavonoids [28,73,74,75]. Considering the potential pharmacological importance of microbial flavonoid metabolites, a deeper understanding of their pharmacokinetic interactions is needed and reasonable. Furthermore, some of the compounds tested also appear in nature and/or are formed during the biotransformation of other polyphenols as well. For example, PHLO derivatives are typically found in plants from the *Myrtacae* and *Rosaceae* families [76], while RES derivatives can be found in rice, wheat, and rye [77].

In conclusion, our results highlight that some microbial metabolites of flavonoids can interact with proteins involved in the pharmacokinetics of drugs. Because many of these metabolites can reach relevant concentrations in the circulation (and likely in some tissues), we should consider the potential interactions of colonic flavonoid metabolites both from the pharmacokinetic and pharmacodynamic points of view. 3CA, 24DHAP, PYR, DESMA, and 2H4MBA form complexes with HSA. However, only slight or no displacement of Site I and II markers was observed in the presence of the compounds tested. Under the applied conditions, DESMA decreased the CYP2C9-catalyzed metabolite formation while colonic metabolites had only a slight effect on the CYP2C19 enzyme. Furthermore, RES and PHLO strongly inhibited CYP3A4. Considering the above-listed observations, besides the flavonoid aglycones and their conjugated derivatives, colonic metabolites may also interfere with the drug therapy through the development of pharmacokinetic interactions.

## Figures and Tables

**Table 1:** Decimal logarithmic values (±SEM) of Stern-Volmer quenching constants (*K_SV_*; unit: L/mol) and binding constants (*K*; unit: L/mol) of albumin-ligand complexes. Means and SEM are derived from three independent experiments. SEM values regarding the fitting to individual data series with Hyperquad software did not exceed 0.02.

Complex	Log*K_SV_*	Log*K*
3CA-HSA	4.13 ± 0.02	4.14 ± 0.03
24DHAP-HSA	4.39 ± 0.06	4.56 ± 0.06
PYR-HSA	4.64 ± 0.03	4.68 ± 0.03
DESMA-HSA	4.99 ± 0.01	5.08 ± 0.06
2H4MBA-HSA	-	5.14 ± 0.06

**Table 2:** Inhibitory effects of DESMA, RES, PHLO, and the positive controls (sulfaphenazole and ketoconazole) on CYP2C9 or CYP3A4 enzymes. Data are derived from three independent experiments.

**CYP2C9 assay**	**Substrate concentration (μM)**	**IC_50_ (μM)**	**α ^1^**
Sulfaphenazole	5.0	1.32	1.00
DESMA	5.0	6.60	5.00
**CYP3A4 assay**	**Substrate concentration (μM)**	**IC_50_ (μM)**	**α ^1^**
Ketoconazole	5.0	0.24	1.00
RES	5.0	4.86	20.3
PHLO	5.0	7.92	33.0

^1^**α**: IC_50_ of the inhibitor divided by the IC_50_ value of the positive control.

## Supplementary Materials

The following are available online at https://www.mdpi.com/2218-273X/10/3/409/s1, Figure S1: Absorption spectra of 3-coumaric acid (3CA, 5 μM), pyrogallol (PYR, 5 μM), 2,4-dihydroxyacetophenone (24DHAP, 5 μM), and O-desmethylangolensin (DESMA, 5 μM) in PBS (pH 7.4).
