# Supplementary material for: Testing the Pharmacokinetic Interactions of 24 Colonic Flavonoid Metabolites with Human Serum Albumin and Cytochrome P450 Enzymes
Source: Biomolecules. 2020 Mar 6;10(3):409. doi: 10.3390/biom10030409 (PMC7175153; doi:10.3390/biom10030409)
Supplement: Supplementary file 1 [file biomolecules-10-00409-s001.pdf]

Supplementary Materials

Testing the Pharmacokinetic Interactions of 24 Colonic Flavonoid Metabolites with Human Serum Albumin and Cytochrome P450 Enzymes

Violetta Mohos [1,2], Eszter Fliszár-Nyúl [1,2], Beáta Lemli [2,3], Balázs Zoltán Zsidó [4], Csaba Hetényi [4], Přemysl Mladěnka [5], Pavel Horký [6,7], Milan Pour [6] and Miklós Poór [1,2,*]

[1] Department of Pharmacology, Faculty of Pharmacy, University of Pécs, Szigeti út 12, Pécs H-7624, Hungary; mohos.violetta@gytk.pte.hu (V.M.); eszter.nyul@aok.pte.hu (E.F.-N.)

[2] János Szentágothai Research Center, University of Pécs, Ifjúság útja 20, Pécs H-7624, Hungary; lemli.beata@gytk.pte.hu (B.L.)

[3] Institute of Organic and Medicinal Chemistry, Medical School, University of Pécs, Szigeti út 12, H-7624 Pécs, Hungary

[4] Department of Pharmacology and Pharmacotherapy, Medical School, University of Pécs, Szigeti út 12, Pécs H-7624, Hungary; zsido.balazs@pte.hu (B.Z.Z.); hetenyi.csaba@pte.hu (C.H.)

[5] Department of Pharmacology and Toxicology, Faculty of Pharmacy in Hradec Králové, Charles University, Heyrovského 1203, 500 05 Hradec Králové, Czech Republic; mladenkap@faf.cuni.cz (P.M.)

[6] Department of Organic and Bioorganic Chemistry, Faculty of Pharmacy in Hradec Králové, Charles University, Akademika Heyrovského 1203, Hradec Králové 500 05, Czech Republic; horkyp@faf.cuni.cz (P.H.); pour@faf.cuni.cz (M.Pour)

[7] Department of Social and Clinical Pharmacy, Faculty of Pharmacy in Hradec Králové, Charles University, Zborovská 2089, Hradec Králové 500 05, Czech Republic

* Correspondence: poor.miklos@pte.hu (M.Poór)

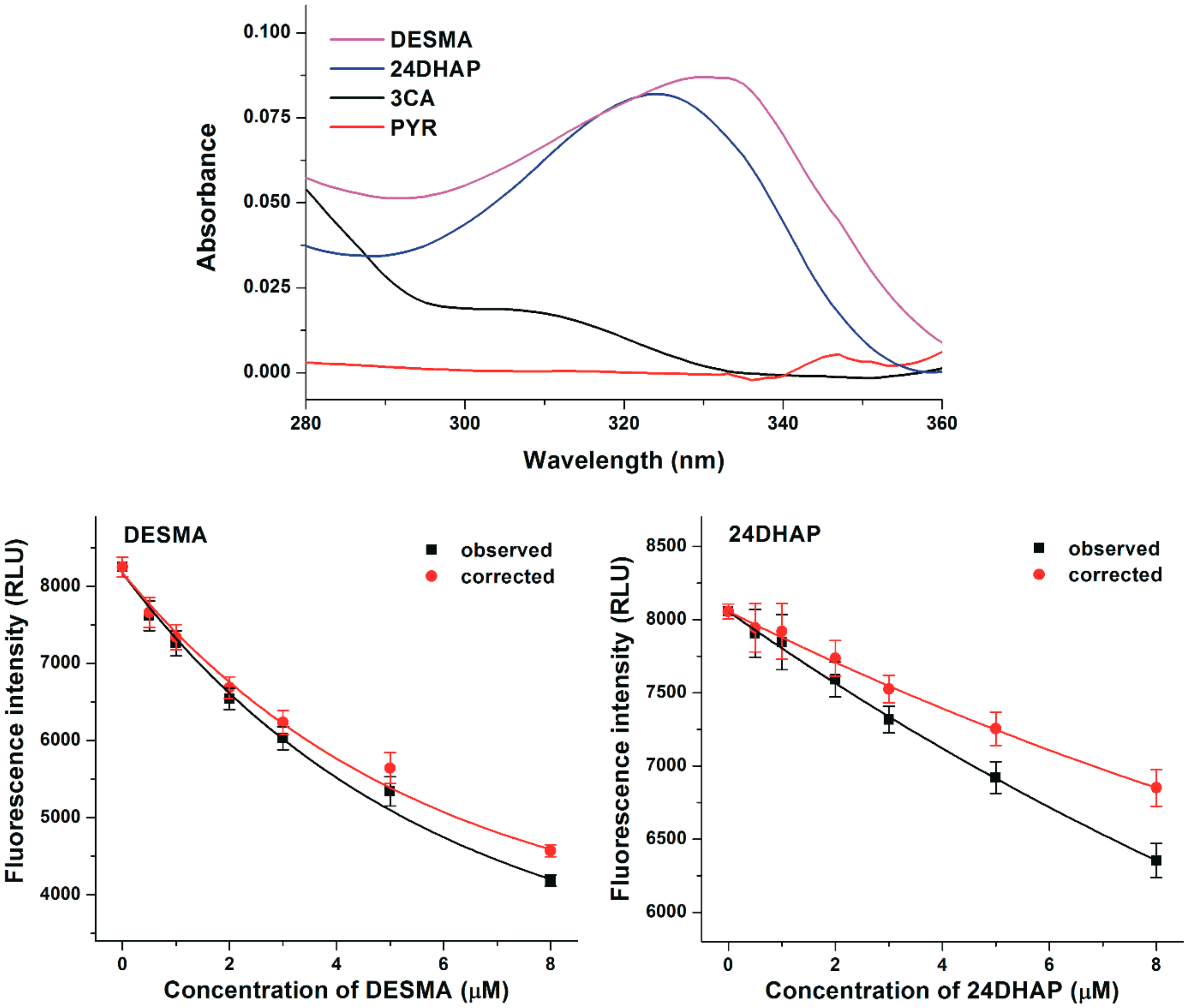

Figure S1. Top: Absorption spectra of 3-coumaric acid (3CA, 5 μM), pyrogallol (PYR, 5 μM), 2,4-dihydroxyacetophenone (24DHAP, 5 μM), and *O*-desmethylangolensin (DESMA, 5 μM) in PBS (pH 7.4). Under the applied conditions, PYR and 3CA showed negligible or low absorption at the wavelengths used in quenching experiments (molar extinction coefficients were the followings: $\varepsilon_{\mathrm{DESMA,\,295\,nm}}$ = 7651 $M^{-1}cm^{-1}$; $\varepsilon_{\mathrm{DESMA,\,340\,nm}}$ = 13179 $M^{-1}cm^{-1}$; $\varepsilon_{\mathrm{24DHAP,\,295\,nm}}$ = 7178 $M^{-1}cm^{-1}$; $\varepsilon_{\mathrm{24DHAP,\,340\,nm}}$ = 8571 $M^{-1}cm^{-1}$; $\varepsilon_{\mathrm{3CA,\,295\,nm}}$ = 4710 $M^{-1}cm^{-1}$; $\varepsilon_{\mathrm{3CA,\,340\,nm}}$ = no absorption; $\varepsilon_{\mathrm{PYR,\,295\,nm}}$ = no absorption; $\varepsilon_{\mathrm{PYR,\,340\,nm}}$ = no absorption). Bottom: Decrease in the fluorescence emission intensity of albumin (2 μM; λ_{ex} = 295 nm, λ_{em} = 340 nm) in the presence of increasing DESMA and 24DHAP concentratrions in PBS (pH 7.4): Observed and corrected (see details in Equation 1) intensities.
